# Ezetimibe: Its Novel Effects on the Prevention and the Treatment of Cholesterol Gallstones and Nonalcoholic Fatty Liver Disease

**DOI:** 10.1155/2012/302847

**Published:** 2011-11-03

**Authors:** Ornella de Bari, Brent A. Neuschwander-Tetri, Min Liu, Piero Portincasa, David Q.-H. Wang

**Affiliations:** ^1^Division of Gastroenterology and Hepatology, Department of Internal Medicine, Edward Doisy Research Center, Saint Louis University School of Medicine, 1100 S. Grand Boulevard, Room 205, St. Louis, MO 63104, USA; ^2^Department of Pathology and Laboratory Medicine, University of Cincinnati College of Medicine, Cincinnati, OH 45237, USA; ^3^Department of Internal Medicine and Public Medicine, Clinica Medica “A. Murri”, University of Bari Medical School, 70124 Bari, Italy

## Abstract

The cholesterol absorption inhibitor ezetimibe can significantly reduce plasma cholesterol concentrations by inhibiting the Niemann-Pick C1-like 1 protein (NPC1L1), an intestinal sterol influx transporter that can actively facilitate the uptake of cholesterol for intestinal absorption. Unexpectedly, ezetimibe treatment also induces a complete resistance to cholesterol gallstone formation and nonalcoholic fatty liver disease (NAFLD) in addition to preventing hypercholesterolemia in mice on a Western diet. Because chylomicrons are the vehicles with which the enterocytes transport cholesterol and fatty acids into the body, ezetimibe could prevent these two most prevalent hepatobiliary diseases possibly through the regulation of chylomicron-derived cholesterol and fatty acid metabolism in the liver. It is highly likely that there is an intestinal and hepatic cross-talk through the chylomicron pathway. Therefore, understanding the molecular mechanisms whereby cholesterol and fatty acids are absorbed from the intestine could offer an efficacious novel approach to the prevention and the treatment of cholesterol gallstones and NAFLD.

## 1. Introduction

The small intestine is a unique organ providing dietary and reabsorbed biliary cholesterol to the body [1–3]. High plasma total and low-density lipoprotein (LDL) cholesterol concentrations are an important risk factor for cardiovascular diseases. The restriction of dietary calories, cholesterol, and saturated fat has been used as the primary initial therapeutic modality for the treatment of patients with dyslipidemia [4]. However, the reduction of dietary cholesterol is frequently not associated with a significant decrease in circulating LDL cholesterol levels, despite significant restrictions in dietary intake. Therefore, pharmacological intervention aimed to reduce intestinal cholesterol absorption is potentially an effective way of lowering plasma total and LDL cholesterol concentrations [2]. The use of cholesterol absorption inhibitors for treating hypercholesterolemia has a long history, and several classes of compounds such as hydrophilic bile acid ursodeoxycholic acid (UDCA) [2], the bile acid sequestrants, specific lipase inhibitors, the intestinal acyl-CoA:cholesterol acyltransferase (ACAT) inhibitors [5, 6], and cholesterol ester transfer protein inhibitors [7] have been developed, and some of them are currently being evaluated in clinical trials. Recently, the discovery and development of ezetimibe, a novel, selective, and potent inhibitor that effectively blocks intestinal absorption of dietary and biliary cholesterol, opened a new door to the treatment of hypercholesterolemia [2, 8–11]. Ezetimibe, which can be administered either as monotherapy or in combination with statins, has been shown to be a safe and efficacious treatment for hypercholesterolemia, potentially enabling more patients to reach recommended LDL cholesterol standards set by the National Cholesterol Education Program Adult Treatment Panel III guidelines [12].

Unexpectedly, it was found that ezetimibe treatment can induce a complete resistance to cholesterol gallstone formation [13] and nonalcoholic fatty liver disease (NAFLD) in addition to its effect on hypercholesterolemia in mice on a Western diet [14]. Furthermore, ezetimibe can prevent gallstones by effectively reducing intestinal cholesterol absorption and biliary cholesterol secretion and protecting gallbladder motility function by desaturating bile in mice. Treatment with ezetimibe also promotes the dissolution of gallstones by forming an abundance of unsaturated micelles in bile. Furthermore, ezetimibe significantly reduces biliary cholesterol saturation and retards cholesterol crystallization in biles of patients with gallstones [15]. It is also found that ezetimibe could prevent fatty liver by reducing hepatic lipogenesis in mice on a high-fat diet and attenuating diet-induced insulin resistance, a state known to drive hepatic lipogenesis through elevated circulating insulin levels [16]. Therefore, it is highly likely that ezetimibe could be a novel approach to reduce biliary cholesterol content and hepatic triglyceride accumulation, and thus a promising strategy for preventing or treating cholesterol gallstones and NAFLD, by inhibiting intestinal cholesterol absorption [15].

In this paper, we will review recent progress in understanding the biochemical and physical-chemical mechanisms, whereby ezetimibe could prevent or treat cholesterol gallstones and NAFLD, the two most prevalent hepatobiliary diseases that constitute a considerable health care burden in the USA.

## 2. Chemistry and Pharmacology of Ezetimibe

Ezetimibe (SCH 58235), 1-(4-fluorophenyl)-(3*R*)-[3-(4-fluorophenyl)-(3*S*)-hydroxypropyl]-(4*S*)-(4-hydroxyphenyl)-2-azetidinone, and an analog, SCH 48461, (3*R*)-(3-Phenylpropyl)-1, (4*S*)-bis(4-methoxyphenyl)-2-azetidinone, are highly selective intestinal cholesterol absorption inhibitors. They can effectively and potently prevent the absorption of cholesterol by inhibiting the uptake and transport of dietary and biliary cholesterol across the apical membrane of enterocytes [17]. It has been found that the 2-azetidinones are able to inhibit cholesterol absorption at very low doses and induce significant reductions in plasma cholesterol concentrations in humans and in a series of different animal models [2, 18–20]. After oral administration, ezetimibe undergoes rapid monoglucuronidation in enterocytes and the liver during its first pass. Because ezetimibe and its glucuronide are enterohepatically recirculated, it is most likely that they could repeatedly produce an inhibitory action on the Niemann-Pick C1-like 1 protein (NPC1L1) on the apical membrane of enterocytes, exhibiting multiple peaks of serum drug concentrations with an elimination half-life of approximately 22 hours [21]. This may explain why ezetimibe has a longer duration of action and why its therapeutic effects persist for several days after its cessation. These observations support the notion that once-daily dosing should be sufficient for an adequate therapeutic effect. It has also been demonstrated that 12 hours after oral administration of the glucuronide (SCH-60663), more than 95% of the compound still can be found in the intestine. Because the glucuronide is more potent in inhibiting cholesterol absorption than ezetimibe, it confirms that ezetimibe acts directly in the intestine as glucuronide [6]. Studies with [^125^I]-labeled ezetimibe glucuronide and [^14^C]-labeled cholesterol have found that the glucuronide could block the cholesterol uptake into the enterocytes [2] because it is often detected in the brush border membrane, a site predominantly associated with cholesterol uptake and transepithelial transport. Furthermore, ezetimibe and its analogs are relatively small molecules that may not be able to change the physical-chemical nature of the intraluminal environment, nor affect the enterohepatic flux of bile acids [2]. 

A careful analysis of 399 patients receiving either placebo- or ezetimibe-doses of 0.25, 1, 5, or 10 mg once daily found a median percentage reduction of plasma LDL cholesterol levels of 0%, 12.7%, 14.7%, 15.8%, and 19.4%, respectively [22]. Ten milligrams of ezetimibe daily reduces intestinal cholesterol absorption by 54% compared with placebo. This effect is accompanied by a decrease in plasma LDL cholesterol levels of 20%, a compensatory increase of 89% in hepatic cholesterol biosynthesis (versus placebo) and is also associated with a decrease in the absorption of plant sterols that are highly structurally related to cholesterol [22]. During ezetimibe treatment, there is a marked compensatory increase in cholesterol biosynthesis in the liver, but not in the extrahepatic organs, and an accelerated loss of cholesterol in the feces with little or no change in the rate of conversion of cholesterol to bile acids. Therefore, the combined administration of the cholesterol absorption inhibitor (ezetimibe) and the 3-hydroxy-3-methylglutaryl coenzyme A (HMG-CoA) reductase inhibitors (e.g., atorvastatin or simvastatin) produces an enhanced reduction in plasma total and LDL cholesterol levels, as well as provides a complementary treatment strategy for high-risk patients, including patients with homozygous familial hypercholesterolemia [23]. These results showed that ezetimibe in combination with HMG-CoA reductase inhibitors would be particularly effective at reducing plasma cholesterol levels in humans. 

In the early studies, it was reported that ezetimibe may not affect intestinal absorption of triglycerides, fatty acids, bile acids, or fat-soluble vitamins, including vitamins A, D, E, and *α*- and *β*-carotenes [24]. More recently, intestinal fatty acid absorption was carefully reexamined by a sensitive and physiologically accurate method, the sucrose polybehenate technique in mice [25, 26]. Instead of monitoring the appearance in plasma of digestion products from an acutely delivered bolus of oil, fecal excretion of dietary fat is measured by this technique and normalized to the excretion of a nonabsorbable fat, sucrose polybehenate, incorporated into the diet [26]. It is observed that on the chow diet, dietary fatty acid absorption is significantly reduced from approximately 95% in control mice to about 87% in ezetimibe-treated mice. Moreover, ezetimibe treatment can significantly reduce intestinal absorption of saturated fatty acids in a graded manner that correlates with chain length. Thus, intestinal absorption of palmitate (16 : 0) and stearate (18 : 0) is reduced from approximately 90% and 70% in control mice to 80% and 50% in ezetimibe-treated mice, respectively. Intestinal absorption efficiency of medium-chain saturated fatty acids is more moderately affected, possibly because medium-chain fatty acid absorption is less dependent on the chylomicron pathway. Myristate (14 : 0) absorption is reduced by 7–10% and laurate (12 : 0) absorption by 4% in ezetimibe-treated mice as compared with control mice [26]. These experiments strongly indicate that ezetimibe can reduce intestinal absorption efficiency of not only cholesterol but also long-chain fatty acids in mice. Additionally, it has been found that besides plasma total and LDL cholesterol concentrations, ezetimibe reduces liver cholesteryl ester levels in a dose-dependent fashion in cholesterol-fed hamsters, rats, and monkeys by inhibiting intestinal cholesterol absorption. However, ezetimibe does not significantly affect plasma HDL cholesterol or triglyceride levels. It was found from an indirect measurement of chylomicrons from plasma, but not lymph, that cynomolgus monkeys fed a single high-cholesterol meal and treated with an ezetimibe analogue displayed a significant reduction in the chylomicron cholesterol content, but not in the triglyceride content [27]. These results suggest that it may be important to carefully investigate the absorption and lymphatic transfer of cholesterol and fatty acids in lymph-fistula animal models. Because chylomicrons and chylomicron remnants may be atherogenic [28], further investigation of this phenomenon might shed more light on the mechanism of the antiatherogenic effect of ezetimibe. It will also be important to investigate whether ezetimibe could influence the lipid and lipoprotein composition of chylomicrons and their physical structure, as well as their assembly and secretion by the enterocytes into the lymph in animals and humans. Of note is that while statins may increase the clearance of chylomicron remnants, they do not reduce the cholesterol content of chylomicrons. Therefore, the combination of a statin and ezetimibe could be highly effective in reducing the atherogenic potential of chylomicrons [29].

## 3. Mechanisms of Ezetimibe Action on Intestinal Absorption of Cholesterol and Fatty Acids

**Figure 1:**
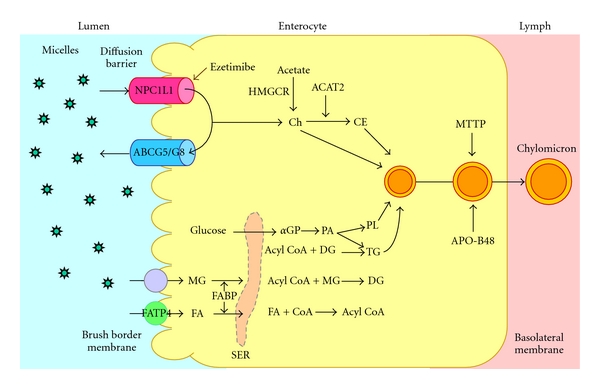
Within the intestinal lumen, the micellar solubilization of sterols facilitates movement through the diffusion barrier overlying the surface of the absorptive cells. In the presence of bile acids, large amounts of the sterol molecules are delivered to the aqueous-membrane interface so that their uptake rate is greatly increased. The Niemann-Pick C1-like 1 protein (NPC1L1), a newly identified sterol influx transporter, is located at the apical membrane of the enterocyte and may actively facilitate the uptake of cholesterol and plant sterols by promoting the passage of these molecules across the brush border membrane of the enterocyte. In contrast, ABCG5/G8 promote active efflux of cholesterol and plant sterols from the enterocyte into the intestinal lumen for excretion. The combined regulatory effects of NPC1L1 and ABCG5/G8 play a critical role in modulating the amount of cholesterol that reaches the lymph from the intestinal lumen. Ezetimibe may reduce cholesterol uptake by the enterocytes through the NPC1L1 pathway, possibly a transporter-facilitated mechanism. Absorbed cholesterol as well as some that is newly synthesized from acetate by 3-hydroxy-3-methylglutaryl-CoA reductase (HMGCR) within the enterocyte is esterified by acyl-CoA:cholesterol acyltransferase isoform 2 (ACAT2) to form cholesteryl esters. It is likely that fatty acids (FA) and monoacylglycerol (MG) could be taken up into enterocytes by facilitated transport. With the assistance of fatty acid binding protein 4 (FABP4), fatty acids and monoacylglycerol are transported into the smooth endoplasmic reticulum (SER) where they are used for the synthesis of diacylglycerol (DG) and then triacylglycerol (TG). Glucose is transported into the SER for the synthesis of phospholipids (PL) through the phosphatidic acid (PA) pathway (abbreviation: *α*-GP, *α*-glycerophosphate). All of these lipids participate in the formation of chylomicrons, a process which also requires the synthesis of apolipoprotein (APO)-B48 and the activity of microsomal triglyceride transfer protein (MTTP). As observed in lymph, the core of the secreted chylomicrons contains triglycerides and cholesteryl esters and the surface of the particles is a monolayer containing phospholipids, mainly phosphatidylcholine, unesterified cholesterol and apolipoproteins including APO-B48, APO-AI, and APO-AIV. Therefore, intestinal cholesterol absorption is a multistep process that is regulated by multiple genes. Reproduced with modifications and with permission from [50].

Although ezetimibe reduces intestinal cholesterol absorption, it does not influence intestinal gene expression levels of *Abcg5*, *Abcg8*, *Sr-b1,* and *Abca1* in mice [2, 30]. Employing a genomic-bioinformatics approach, Altmann et al. [31] identified transcripts containing expression patterns and structural characteristics anticipated in cholesterol transporters (e.g., sterol-sensing and transmembrane domains, extracellular signal peptides) and established a strong candidate for the ezetimibe-sensitive cholesterol transporter, the awkwardly named Niemann-Pick C1-like protein 1 (NPC1L1). NPC1L1 has 50% amino acid homology to NPC1 [31], which is defective in the cholesterol storage disease Niemann-Pick type C and functions in intracellular cholesterol trafficking [32]. However, in contrast to *NPC1* that is expressed in many tissues [33, 34], *NPC1L1* is expressed predominantly in the gastrointestinal tract with peak expression in the proximal jejunum. Subfractionation of brush border membranes suggests that NPC1L1 is associated with the apical membrane fraction of enterocytes. Moreover, NPC1L1 deficient mice show a ~65% reduction in intestinal cholesterol absorption (16%) compared with wild-type mice (45%). The cholesterol absorption efficiency in NPC1L1 deficient mice is unaffected by ezetimibe or cholic acid, supporting the presence of redundant alternative pathways [31]. These studies strongly suggest that NPC1L1 could be an ezetimibe-sensitive target protein and is responsible for cholesterol uptake by the enterocyte for intestinal absorption (Figure 1) [31].

Many of the details of how ezetimibe prevents cholesterol absorption have been elucidated, and recently a molecular mechanism for cholesterol uptake mediated by the NPC1L1 has been proposed. The NPC1L1 protein recycles between the plasma membrane facing the extracellular space and the endocytic recycling compartment [35]. If the cholesterol concentration in the intestinal lumen is high, it is incorporated into the plasma membrane and is sensed by NPC1L1 that is localized on the surface of apical membrane of the enterocytes [34]. Both NPC1L1 and cholesterol are then internalized together through clathrin/AP2-mediated endocytosis [36]. The clathrin-coated globular vesicles are transported along microfilaments to the endocytic recycling compartment where large quantities of cholesterol and NPC1L1 are subsequently stored [36, 37]. If the intracellular cholesterol level is low, endocytic recycling compartment-localized NPC1L1 free of cholesterol moves back to the plasma membrane along microfilaments to transfer new cholesterol as it is absorbed by the enterocytes. The key role of the NPC1L1 inhibitor ezetimibe is to prevent NPC1L1 from entering the AP2-mediated clathrin-coated vesicles. At this stage, the endocytosis of NPC1L1 is inhibited and cholesterol absorption is decreased [36].

Although it has been observed that ezetimibe can reduce intestinal fatty acid absorption in mice, the molecular mechanism of this action is still unclear. As reviewed above, the deletion of the *Npc1l1* gene also reduces intestinal absorption of fatty acids, especially long-chain fatty acids. A potential mechanism may be that inhibition of intestinal cholesterol absorption by ezetimibe could somehow influence intestinal expression of genes involved in fatty acid uptake and transport. It is well known that hydrolysis and absorption of dietary fat (mainly triglycerides) are extremely efficient processes (>90%). However, it remains a matter of debate whether intestinal fatty acid absorption occurs solely by passive diffusion or also by protein-facilitated transport. Some studies have suggested that fatty acid transporter/cluster determinant 36 (FAT/CD36) may play a role in intestinal fatty acid absorption [38, 39]. Thus, it has been hypothesized that ezetimibe may have potential inhibitory effects on “protein-facilitated” absorption of fatty acids by enterocytes [26]. As found by Western blot analysis, protein concentrations of fatty acid transport protein 4 (FATP4) in the small intestine are significantly reduced by approximately 50% in ezetimibe-treated mice compared with control mice (Figure 1), which is associated with reduced intestinal absorption of long-chain saturated fatty acids [26, 40]. It is unclear whether this inhibitory effect on intestinal FATP4 is induced by ezetimibe through a direct or indirect action pathway. Another explanation is that ezetimibe treatment significantly reduces cholesterol absorption so that the physical structure of chylomicrons may be modified and their assembly, and/or secretion into the lymphatics may be impaired. Because chylomicrons are a crucial vehicle for the transfer of cholesterol and fatty acids as triglyceride from the intestinal lumen to the lymph, impairing their formation by reducing cholesterol availability may induce a secondary action on fatty acid absorption. Because of this possible mechanism of action, it will be important to examine the physical structure of chylomicrons and their assembly and secretion into lymph to prove this hypothesis.

## 4. Physical-Chemistry of Bile, Physical Forms of Cholesterol Carrier, and Pathophysiology of Cholesterol Gallstones

Cholesterol, phospholipids, and bile salts are three major lipid components of bile in animals and humans [41]. Because cholesterol is virtually insoluble in an aqueous medium such as bile, specialized transport mechanisms are required to maintain it in solution and the mechanism for its solubilization in bile is complex. Similarly, phospholipids are insoluble in water and require carrier vehicles in bile. Bile salts have the property of amphiphilicity with both hydrophilic and hydrophobic areas of the molecules and are soluble in aqueous solutions to varying degrees, depending on the number and characteristics of hydroxyl groups and side chains, as well as the composition of the particular aqueous solution. Bile salt monomers can aggregate spontaneously to form simple micelles when their concentration exceeds the critical micellar concentration [42]. As defined, a micelle is a colloidal aggregation of molecules of an amphipathic compound (e.g., bile salt) in which the hydrophobic portion of each molecule faces inward and the hydrophilic groups point outward [41]. The formation of simple micelles of bile salts alone depends primarily on the concentration of bile salts. Thus, micelles are formed at, but not below, a critical micellar concentration of bile salts in bile, which is approximately 2 mmol/L [41]. The formation of micelles is also influenced by the concentrations of biliary solids and counterions, by the type of bile salt (i.e., by its degree of hydroxylation and whether it is conjugated with taurine or glycine or not), and by the temperature and pH of the bile. These simple micelles (~3 nm in diameter) are small, thermodynamically stable aggregates that are principally composed of bile salts [43]. The cholesterol can be solubilized within the hydrophobic center of the micelle. Also, simple micelles of bile salts are capable of solubilizing and incorporating phospholipids. This enables the micelles—then referred to as mixed micelles—to solubilize at least three times the amount of cholesterol solubilized by simple micelles. The solubility of cholesterol in mixed micelles is enhanced when the concentration of total lipids (bile salts, phospholipids, and cholesterol) in bile is high. Moreover, maximal solubility occurs when the molar ratio of phospholipids to bile salts is between 0.2 and 0.3 [41]. Mixed micelles (~4–8 nm in diameter) are large, thermodynamically stable aggregates that are composed of bile salts, cholesterol, and phospholipids. Their size varies depending on the relative proportion of bile salts and phospholipids. The shape of a mixed micelle is that of a lipid bilayer with the hydrophilic groups of the bile salts and phospholipids aligned on the “outside” of the bilayer, interfacing with the aqueous bile, and the hydrophobic groups on the “inside.” Cholesterol molecules can, therefore, be solubilized on the inside of the bilayer away from the aqueous areas on the outside. The amount of cholesterol that can be solubilized in micelles depends on the relative proportions of bile salts and phospholipids, with additional phospholipids aiding in cholesterol solubilization [41].

Studies using techniques such as quasielastic light-scattering spectroscopy (QLS) and electron microscopy to investigate the physical-chemistry of model and native bile samples have defined more complex mechanisms of cholesterol solubilization in bile [41, 44, 45]. Beside simple and mixed micelles, biliary vesicles, nonmicellar carriers of cholesterol, do exist in bile for the solubilization of cholesterol. Vesicles are unilamellar spherical structures and contain phospholipids, cholesterol, and little, if any, bile salts. Thus, vesicles (~40 to 100 nm in diameter) are substantially larger than either simple or mixed micelles, but much smaller than liquid crystals (~500 nm in diameter) that are composed of multilamellar spherical structures. Vesicles are present in large quantities in hepatic bile and are presumably secreted by the hepatocyte [41].

Vesicles in bile have one of two distinct origins. Those formed at the canalicular membrane of hepatocytes are unilamellar and rich in phosphatidylcholines compared with cholesterol (i.e., contain one cholesterol molecule per three phosphatidylcholine molecules). Because of increasing bile salt concentrations in the biliary tree, these vesicles rapidly undergo structural rearrangements and are therefore detectable only in bile specimens analyzed immediately after collection. A second type of vesicle forms spontaneously in bile when the capacity of mixed and simple micelles to solubilize cholesterol is exceeded. These unilamellar or multilamellar vesicles are cholesterol-rich, with cholesterol content reaching as high as two cholesterol molecules per phosphatidylcholine molecule. 

When concentrations of bile salts are relatively low, vesicles are relatively stable, especially in dilute hepatic bile. Moreover, vesicles may transform or convert completely to mixed micelles when bile salt concentrations in concentrated gallbladder bile are increased. When the bile salt concentration is not high enough, only some vesicles convert to micelles. Because relatively more phospholipids than cholesterol can be transferred from vesicles to mixed micelles, the residual vesicles, now remodeled, may be rich in cholesterol relative to the phospholipids. If the remaining vesicles have a relatively low cholesterol/phospholipid ratio (<1), they are relatively stable. However, if the cholesterol/phospholipid ratio in vesicles is >1, vesicles become increasingly unstable [46]. These cholesterol-rich vesicles may transfer some cholesterol to less cholesterol-rich vesicles or to micelles, or may fuse or aggregate to form larger (~500 nm in diameter) multilamellar vesicles that may now be termed liposomes or liquid crystals [41]. Liquid crystals are visible by polarizing light microscopy as lipid droplets with birefringence in the shape of a Maltese cross. Liquid crystals are inherently unstable and may form solid cholesterol monohydrate crystals, which is termed cholesterol nucleation. As a result, the nucleation of cholesterol monohydrate crystal induces a decrease in the amount of cholesterol contained in vesicles but not of cholesterol in micelles, supporting the concept that vesicles could serve as the primary source of cholesterol during cholesterol nucleation and crystallization [41].

**Figure 2:**
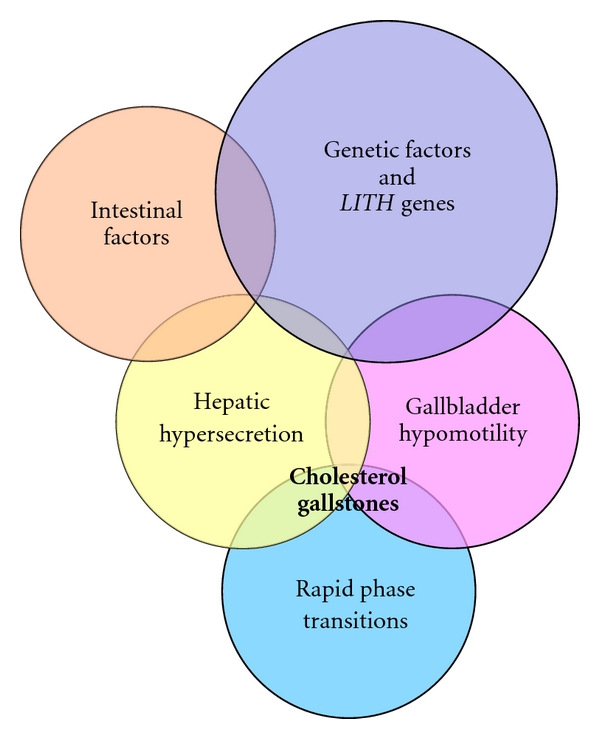
Venn diagram of five primary defects: genetic factors and *LITH* genes, hepatic hypersecretion, gallbladder hypomotility, rapid phase transitions, and intestinal factors. The hypothesis proposed is that hepatic cholesterol hypersecretion into bile is the primary defect and is the outcome in part of a complex genetic predisposition. The downstream effects include gallbladder hypomotility and rapid phase transitions. A major result of gallbladder hypomotility is alteration in the kinetics of the enterohepatic circulation of bile salts, resulting in increased cholesterol absorption and reduced bile salt absorption that lead to abnormal enterohepatic circulation of bile salts and diminished biliary bile salt pool size. Not only does gallbladder hypomotility facilitate cholesterol nucleation/crystallization, but also it allows the gallbladder to retain cholesterol monohydrate crystals. Although a large number of candidate *Lith* genes have been identified in mouse models, the identification of human *LITH* genes and their contributions to gallstones require further investigation. Reproduced with modifications and with permission from [48].

It is well known that cholesterol cholelithiasis is a multifactorial disease influenced by a complex interaction of genetic and environmental factors [15, 42, 47]. Based on recent studies on humans and mouse models, a novel concept has been proposed that interactions of five defects could play an important role in determining the formation of cholesterol gallstones (Figure 2), which are considered in terms of *LITH* genes (genetic defect), thermodynamics (solubility defect), kinetics (nucleation defect), stasis (residence time defect), and lipid sources (metabolic defect) [48]. Furthermore, cholesterol gallstone formation represents a failure of biliary cholesterol homeostasis in which the physical-chemical balance of cholesterol solubility in bile is disturbed [41, 42, 47]. The liver is the source of cholesterol-supersaturated bile in the gallbladder with cholesterol gallstones. Thus, gallstones can be viewed in one sense as a liver disease because some metabolic defects or a combination of defects within the liver result in hypersecretion of biliary cholesterol. As noted, supersaturated bile is a prerequisite for cholesterol gallstone formation, and hypersecretion of biliary cholesterol is the primary metabolic abnormality responsible for initiating cholelithiasis. However, the gallbladder and intestine also conspire as part of a “vicious cycle” that creates physical-chemical instabilities in bile and culminates in the formation of cholesterol gallstones. Therefore, the formation of cholesterol gallstones is the final consequence of excess secretion of cholesterol from the liver into bile [42, 49]. It has been hypothesized that reducing cholesterol bioavailability in the liver for biliary secretion can prevent the formation of cholesterol gallstones and promote the dissolution of cholesterol crystals and gallstones. This information on the physical-chemistry of bile and the physical forms of cholesterol carriers can help us in understanding why ezetimibe could have a potential therapeutic effect on cholesterol gallstones.

## 5. Effects of Ezetimibe on the Prevention and the Treatment of Cholesterol Gallstones

Although some, but not all, studies found that high dietary cholesterol is associated with increased hepatic secretion of biliary cholesterol, epidemiological investigations have clearly demonstrated that cholesterol cholelithiasis is prevalent in cultures consuming a “Western” diet consisting of high total calories, cholesterol, saturated fatty acids, refined carbohydrates, proteins, and salt, as well as low fiber content. Many studies have found that the gallstone incidence in North and South American as well as European populations is significantly higher than that in Asian and African populations [15, 53, 54]. Furthermore, several clinical studies have found an association between the increased incidence of cholesterol gallstones in China and a “westernization” of the traditional Chinese diet. In Japan, cholesterol cholelithiasis once was rare, but over the past 40 years with the adoption of Western-type dietary habits, the incidence has increased markedly [15, 55, 56]. Moreover, it has been observed that there is a significant and positive correlation between the efficiency of intestinal cholesterol absorption and the prevalence of cholesterol gallstone formation in mice, suggesting that high efficiency of intestinal cholesterol absorption and high dietary cholesterol are two independent risk factors in the formation of cholesterol gallstones [57]. In addition, in mouse studies, targeted deletion of the acyl-CoA:cholesterol acyltransferase gene 2 (*Acat2*) resulted in the lack of cholesterol ester synthesis in the small intestine. This causes a marked reduction in intestinal cholesterol absorption and a complete resistance to diet-induced cholesterol gallstones [13, 15]. Furthermore, the absence of expression of intestinal APO-B48, but not APO-B100, reduces biliary cholesterol secretion and cholelithogenesis, possibly by decreasing intestinal absorption and hepatic bioavailability [15, 58]. Reduced gallstone prevalence in lithogenic diet-fed apolipoprotein E knockout mice may be explained by decreased availability of chylomicron-derived cholesterol in the liver for biliary secretion [15, 59]. These studies support the notion that high dietary cholesterol through the chylomicron pathway could provide an important source of excess cholesterol molecules for secretion into bile, thereby inducing cholesterol-supersaturated bile and enhancing cholelithogenesis [15].

Indeed, because biliary cholesterol hypersecretion is an important prerequisite for cholesterol gallstone formation [15, 42, 47], inhibition of cholesterol absorption in the intestine, or hepatic uptake of chylomicron remnants has become an attractive alternative to decrease biliary cholesterol secretion and saturation [15]. Since ezetimibe significantly suppresses intestinal cholesterol absorption via the NPC1L1 pathway [15, 60], possibly a transporter-facilitated mechanism [15, 34], this should reduce the cholesterol content of the liver, which in turn decreases bioavailability of cholesterol for biliary secretion [15]. 

**Figure 3:**
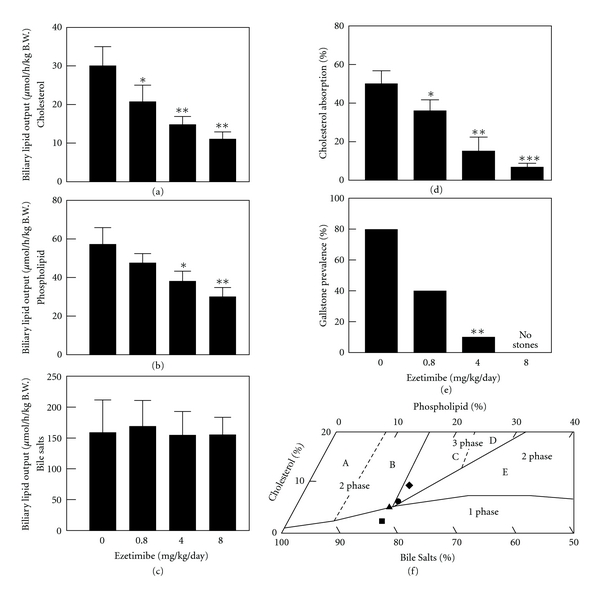
Effect of ezetimibe on the prevention of cholesterol gallstones. Ezetimibe significantly reduced, in a dose-dependent fashion, hepatic output of (a) biliary cholesterol and (b) phospholipid, but not (c) bile salts. *P < 0.05, **P < 0.01, and ***P < 0.001, compared with mice fed the lithogenic diet and receiving no ezetimibe. (d) There is a clear dose-dependent reduction in intestinal cholesterol absorption efficiency from 50 ± 6% to 4 ± 2% in chow-fed with mice, as measured by the fecal dual-isotope ratio method. (e) When doses of ezetimibe are increased from 0 to 4 mg/kg/day, gallstone prevalence rates are reduced from 80% to 10% in mice fed with the lithogenic diet for 8 weeks. No gallstones are found in mice treated with ezetimibe at 8 mg/kg/day. (f) The relative lipid composition of pooled gallbladder bile from mice fed with the lithogenic diet and receiving no ezetimibe are located in the central three-phase zone, where bile is composed of solid cholesterol monohydrate crystals, liquid crystals, and saturated micelles at equilibrium. In contrast, administration of the highest dose (8 mg/kg/day) of ezetimibe resulted in the relative biliary lipid composition of pooled gallbladder bile plotted in the one-phase micellar zone, even upon the lithogenic diet feeding for 8 weeks. By phase analysis, these bile samples are composed of unsaturated micelles at equilibrium. A symbol ♦ represents relative lipid composition of pooled gallbladder bile at 8 weeks on the lithogenic diet supplemented with ezetimibe at 0; ● 0.8; ▲ 4; ■ 8 mg/kg/day. Reproduced with modifications and with permission from [15].

**Figure 4:**
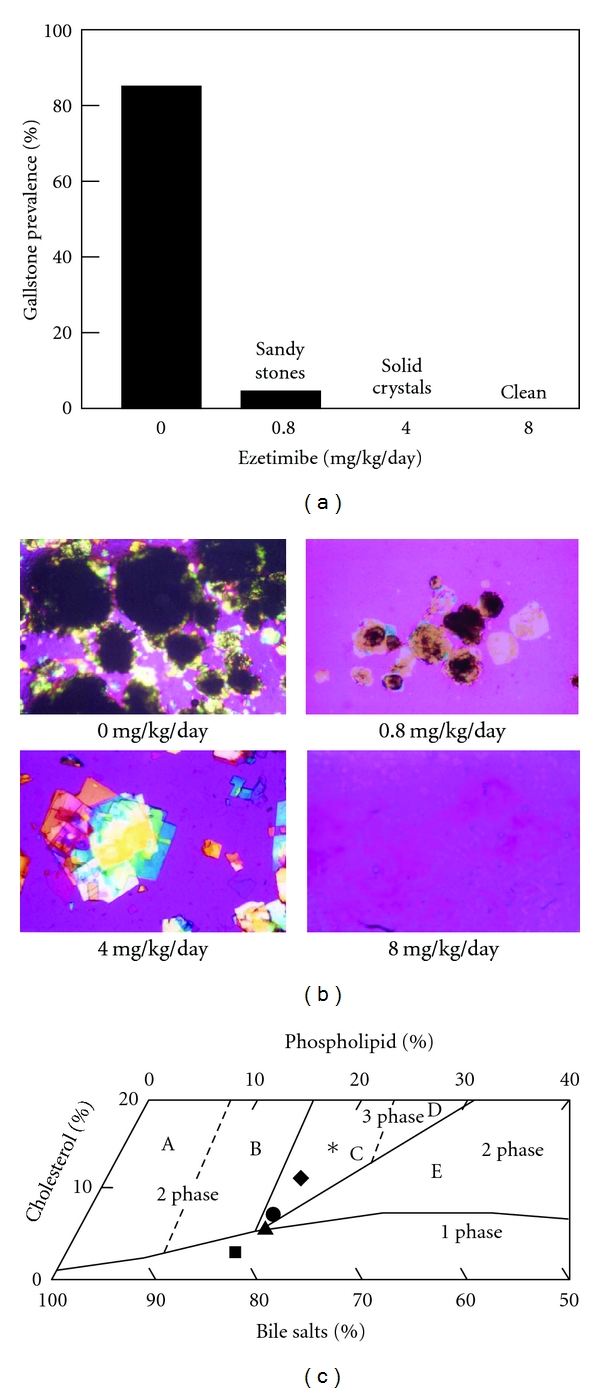
Effect of ezetimibe on the dissolution of cholesterol gallstones. (a) For gallstone dissolution experiments, mice with preexisting gallstones were fed a chow diet alone for 8 weeks, which does not result in a spontaneous dissolution of gallstones. In contrast, treatment with ezetimibe at 0.8 to 8 mg/kg/day induces rapid dissolution of gallstones. Gallstones were completely dissolved by the highest (8 mg/kg/day) dose of ezetimibe. (b) Representative photomicrographs of mucin gel, liquid crystals, cholesterol monohydrate crystals, and gallstones as observed in gallbladder biles at week 8 after ezetimibe treatment. All magnifications are ×800, except for ezetimibe treatment at 0 and 0.8 mg/kg/day, which are ×400, by polarizing light microscopy. (c) The relative lipid composition of pooled gallbladder bile from mice fed 8 weeks with the chow diet supplemented with varying doses of ezetimibe is plotted on a condensed phase diagram. Because of a 12-week feeding period of the lithogenic diet, the relative lipid composition of pooled gallbladder bile from mice that have formed cholesterol gallstones is located in the central three-phase zone. Although the lithogenic diet is replaced with the chow diet for 8 weeks, the relative biliary lipid composition of bile is still in region C, where at equilibrium the bile is composed of solid cholesterol crystals, liquid crystals, and saturated micelles. By feeding varying doses of ezetimibe, the relative lipid composition of pooled gallbladder bile gradually shifts down and, finally, enters the one-phase micellar zone. These alterations explain that gallstones are dissolved through an unsaturated micelle mechanism. A symbol ∗ represents relative lipid composition of pooled gallbladder bile from mice that have preexisting gallstones and before ezetimibe treatment; ♦ relative lipid composition of pooled gallbladder bile at the end of the gallstone dissolution study at week 8 of feeding the chow diet only (control); ● 0.8; ▲ 4; ■ 8 mg/kg/day of ezetimibe. Reproduced with modifications and with permission from [15].

It has been found that ezetimibe induces a significant dose-dependent reduction in intestinal cholesterol absorption efficiency, coupled with a significant dose-dependent decrease in biliary cholesterol outputs and gallstone prevalence rates (Figure 3). In particular, even under high dietary cholesterol loads, cholesterol gallstones can be prevented by ezetimibe in C57L mice carrying the *Lith1* and *Lith2* genes that predispose to cholesterol stone formation [15]. Although ezetimibe substantially reduces cholesterol concentrations and to some extent phospholipid concentrations, but not bile acid concentrations in gallbladder bile, all crystallization pathways and phase boundaries on the bile phase diagram are not influenced by ezetimibe [15]. Furthermore, in company with increased doses of ezetimibe, the relative lipid compositions of pooled gallbladder bile samples are progressively shifted down and to the left of the phase diagram, entering the one-phase micellar zone where there is an abundance of unsaturated micelles, but never solid cholesterol crystals or liquid crystals. Because the micellar cholesterol solubility is dramatically increased in gallbladder bile, the cholesterol molecules can be transferred from the cholesterol monohydrate surface into unsaturated micelles. As a result, gallstones become smaller and eventually dissolved (Figure 4) [15]. This excellent physical-chemical mechanism could explain, in part, how ezetimibe treatment prevents cholesterol gallstone formation in mice.

Enlarged fasting gallbladder volume, together with impaired postprandial and interdigestive gallbladder emptying, is a frequent and distinctive feature in gallstone patients [15, 61, 62], indicating that the gallbladder is another key player in cholelithogenesis. This type of “gallbladder stasis” provides time for nucleation of cholesterol crystals and their aggregation into macroscopic stones [15, 42, 47, 62]. Under conditions of cholesterol-supersaturated bile, the gallbladder absorbs a large amount of cholesterol, thereby resulting in the accumulation of excess cholesterol in the gallbladder wall. Because gallbladder absorptive cells apparently cannot assemble lipoproteins for lipid transport into plasma, the absorbed cholesterol is converted to cholesteryl ester and stored in the mucosa and lamina propria. As a result, excess cholesterol in smooth muscle cells could stiffen sarcolemmal membranes and decouple the G-protein-mediated signal transduction that usually occurs when CCK binds to its receptor, thereby further paralyzing gallbladder contractile function and consequently impairing gallbladder emptying function. These lithogenic effects on gallbladder motility function can be completely inhibited by ezetimibe [15, 63]. This effect of ezetimibe on protecting gallbladder motility can mostly be attributed to the desaturation of bile. 

Ursodeoxycholic acid (UDCA) is currently used as a first-line pharmacological therapy to treat only a subgroup of symptomatic patients with small, radiolucent cholesterol gallstones [15, 47, 64]. Extensive clinical studies have shown that long-term administration of UDCA promotes the dissolution of cholesterol gallstones and prevents the recurrence of gallstones after extracorporeal shock wave lithotripsy [15, 65]. However, because of a failure to titrate the dose adequately, optimal use of UDCA is not always achieved in clinical practice [15]. It should be pointed out that the hydrophilic bile acid UDCA can greatly favor the formation of vesicles in bile, which can enhance the growth of liquid crystals on the cholesterol monohydrate surface and their subsequent dispersion might occur during gallstone dissolution. Consequently, liquid crystalline dissolution allows the transport of a great amount of cholesterol from stones [15]. Because the cholelitholytic mechanism of ezetimibe is totally different from that of hydrophilic bile acids such as UDCA, it has been proposed that a combined therapy of ezetimibe and UDCA could be a faster means to promote the dissolution of cholesterol gallstones, because of the two distinct mechanisms via the formation of unsaturated micelles by ezetimibe and a liquid crystalline mesophase by UDCA [15], respectively.

A clinical study has been performed to examine whether ezetimibe would reduce biliary cholesterol concentrations in gallstone patients compared to overweight subjects without gallstones [15]. It was observed that 30 days after starting the medication, ezetimibe at 20 mg/day significantly reduced cholesterol concentrations and cholesterol saturation indexes (CSIs) of gallbladder biles in gallstone patients (Table 1), similar to the results as observed in mouse studies [15]. Consequently, cholesterol crystallization was retarded and detection time of cholesterol monohydrate crystals was significantly delayed as analyzed by polarized light microscopy. Although similar results between mice and gallstone patients have been observed regarding the effect of ezetimibe on the reduction in bile cholesterol concentrations and cholesterol crystallization, a long-term human study is needed to observe whether ezetimibe can reduce gallstone prevalence and completely dissolve gallstones [15]. 

It should be emphasized that there is a difference in tissue distribution of NPC1L1 between mice and humans. In mice, NPC1L1 is expressed only in the intestine, while in humans, it can be detected in both the intestine and liver [34]. Because of this, it has been hypothesized that ezetimibe may have different effects on biliary cholesterol output in humans than in mice. It has been found that the secretion efficiency of biliary cholesterol is most likely determined by the net effect between efflux and influx of cholesterol molecules across the canalicular membrane of the hepatocyte, which could be regulated by the ABCG5/G8-dependent and independent pathways as well as the NPC1L1 pathway [15, 58]. Indeed, ezetimibe treatment can reduce bile cholesterol content and CSIs and prolong detection times of cholesterol monohydrate crystals in humans. One possible reason for these results in humans is that because biliary cholesterol secretion is a unique path for excretion of cholesterol from the body in humans and animals, hepatic ABCG5/G8 may play a stronger role in the regulation of biliary cholesterol secretion than NPC1L1. Another possible explanation is that in the gut-liver axis, the intestinal NPC1L1 may play a significant role in providing dietary and reabsorbed biliary cholesterol to the body, and the inhibition of its functions by ezetimibe significantly reduces cholesterol absorption. So, the bioavailability of cholesterol from intestinal sources for biliary secretion is decreased markedly. In contrast, inhibition of the hepatic NPC1L1 by ezetimibe has a weak effect on biliary cholesterol secretion and CSI values [15]. More interestingly, similar to humans, the Golden Syrian hamster displays the abundance of NPC1L1 in the small intestine that far exceeds that in other regions of the gastrointestinal tract such as liver and gallbladder [66]. The tissue distribution pattern of NPC1L1 is nearly similar between hamsters and humans. It was found that the ezetimibe-induced reduction in intestinal cholesterol absorption is coupled with a decrease in the absolute and relative cholesterol levels in bile in hamsters fed a high-cholesterol diet [66]. These results are consistent with a recent finding that ezetimibe treatment significantly reduces biliary cholesterol saturation in patients with gallstones. 

**Figure 5:**
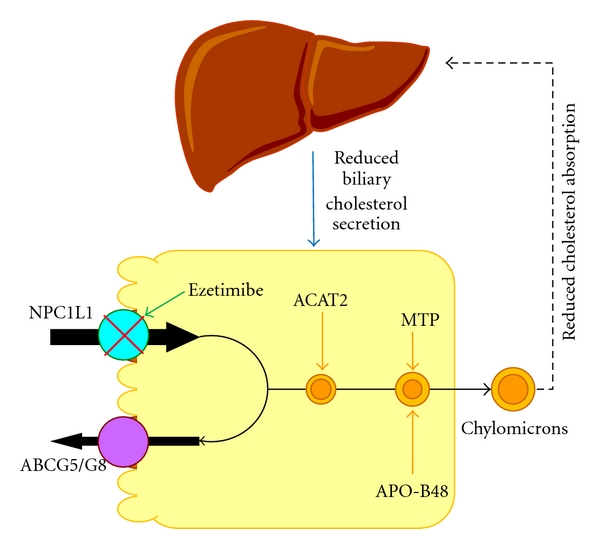
Pathways underlying the absorption of cholesterol from the intestinal lumen and its delivery to the liver. High dietary cholesterol delivery through the chylomicron pathway could provide an important source of excess cholesterol molecules for hepatic secretion into bile, thereby inducing cholesterol-supersaturated bile and enhancing cholesterol gallstone formation. Ezetimibe significantly suppresses cholesterol absorption from the small intestine via the Niemann-Pick C1-like 1 (NPC1L1) pathway, possibly by a transporter-facilitated mechanism. This effect of ezetimibe could significantly diminish the cholesterol content of the liver, which in turn remarkably decreases bioavailability of cholesterol for hepatic secretion into bile. ABCG5/G8: ATP-binding cassette (transporters) G5 and G8; ACAT2: acyl-CoA:cholesterol acyltransferase isoform 2; APO-B48: apolipoprotein B48; MTTP: microsomal triglyceride transfer protein. See text for details.

Overall, ezetimibe treatment can prevent cholesterol gallstones mainly through inhibiting intestinal cholesterol absorption so that hepatic secretion of biliary cholesterol is reduced, and gallbladder motility function is preserved by desaturating bile (Figure 5). Also, ezetimibe promotes the dissolution of cholesterol gallstones through a greater capacity to form an abundance of unsaturated micelles. Therefore, ezetimibe is a novel and potential cholelitholytic agent for both preventing and treating cholesterol gallstones [15]. 

## 6. Pathophysiology of Nonalcoholic Fatty Liver Disease (NAFLD)

Nonalcoholic fatty liver disease (NAFLD) is a chronic liver disease, which includes a spectrum of hepatic pathology ranging from simple triglyceride accumulation in hepatocytes (hepatic steatosis) to hepatic steatosis with inflammation (steatohepatitis), fibrosis, and cirrhosis in the absence of alcohol abuse and other causes [67–69]. NAFLD is characterized pathologically by macrovesicular steatosis, mild diffuse lobular mixed acute and chronic inflammation, perivenular and zone 3 perisinusoidal collagen deposition, hepatocyte ballooning, poorly formed Mallory-Denk bodies, glycogen nuclei in periportal hepatocytes, lobular lipogranulomas, and PAS-diastase-resistant Kupffer cells [70, 71]. 

NAFLD was once proposed to be the result of two distinct but related “hits” to the hepatocyte [72, 73]. The first “hit” is the development of lipid accumulation and hepatic steatosis because of an imbalance of hepatic lipid metabolism, which leads to either excessive lipid influx, decreased lipid clearance, or both [70]. At this point, steatosis is potentially reversible and does not necessarily induce permanent hepatic injury. Although it is less common and occurs in approximately 5% of individuals with steatosis, the second “hit” is more virulent, being an inflammatory process that is induced probably by oxidative stress, lipid peroxidation, and cytokine action [74]. The resulting lobular inflammation causes ballooning degeneration and perisinusoidal fibrosis, which promote apoptosis, and hepatocellular death. These alterations eventually induce scarring and progression to nonalcoholic steatohepatitis (NASH) [75]. However, many studies have been unable to prove that either oxidant stress or lipid peroxidation is necessary for the development of steatohepatitis in humans. 

**Figure 6:**
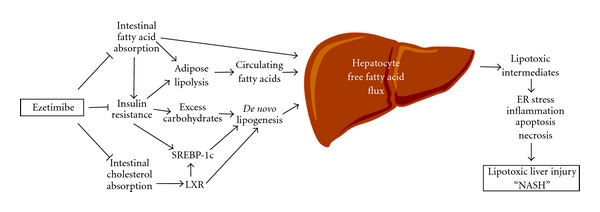
Potential therapeutic effects of ezetimibe on nonalcoholic fatty liver disease and steatohepatitis (NAFLD and NASH). On the basis of the lipotoxicity model of NAFLD and NASH [51], it has been proposed that metabolites of unesterified fatty acids may induce lipotoxic hepatocellular injury manifested as ER stress, inflammation, apoptosis, necrosis, and dysmorphic features such as ballooning and Mallory-Denk body formation. The generation of lipotoxic metabolites of fatty acids often takes place in parallel with the accumulation of triglyceride droplets (steatosis) in the liver. A high-fat diet often causes insulin resistance, a state that is associated with hyperinsulinemia and hyperglycemia. Because insulin resistance promotes an excessive flow of fatty acids from adipose tissue and also impairs peripheral glucose disposal, these alterations increase the need for fatty acid disposal in the liver through oxidative pathways and through the formation of triglyceride which is then either stored temporarily as lipid droplets or secreted as VLDL. Furthermore, elevated blood insulin and glucose activate transcription factors SREBP-1c to increase hepatic lipogenic gene expression. In addition, intestinal cholesterol absorption promotes hepatic lipogenesis via cholesterol-dependent activation of LXR. Ezetimibe treatment could block (i) intestinal fatty acid absorption, which could reduce a delivery of fatty acids from the gut to the adipose tissue through the chylomicron pathway; (ii) diet-induced insulin resistance in part by reducing intestinal fatty acid absorption; (iii) cholesterol-driven lipogenesis by inhibiting intestinal cholesterol absorption, which together may substantially reduce the burden of fatty acids on the liver. ER: endoplasmic reticulum; LXR: liver X receptor; SREBP-1c: sterol regulatory element-binding protein-1c.

Recently, the lipotoxicity model of NASH pathogenesis has emerged based on evidence showing that triglyceride often accumulates in the liver as a parallel rather than pathogenic process during lipotoxic hepatocellular injury (Figure 6) [51]. Thus, it has been hypothesized that metabolites of unesterified fatty acids play a critical role in inducing lipotoxic injury in the liver. The generation of lipotoxic metabolites of fatty acids typically occurs in parallel with the accumulation of triglyceride droplets (steatosis), resulting in a phenotype recognized as NASH, where steatosis and features of cellular injury are present together [51]. Metabolic abnormalities predisposing to lipotoxic injury include an increased supply or impaired disposal of unesterified fatty acids. More importantly, insulin resistance could play a central role in these processes by allowing unsuppressed lipolysis in adipocytes resulting in an excessive flow of fatty acids from adipose tissues and also impairing peripheral glucose disposal [51]. *De novo* lipogenesis in the liver using excessive dietary carbohydrate as a substrate for fatty acid synthesis is also a significant contributor to the burden of saturated fatty acids in the liver. Fatty acid disposal in the liver occurs through oxidative pathways and through the formation of triglyceride which is either stored temporarily as lipid droplets or secreted as VLDL [51]. Additional factors, including oxidative stress, mitochondrial dysfunction, gut-derived lipopolysaccharide and adipocytokines, may promote further hepatocellular damage [76, 77]. These processes can lead to inflammation, necrosis, apoptosis and fibrogenesis, which may ultimately lead to cirrhosis, liver failure, hepatocellular carcinoma and death [78].

## 7. Potential Therapeutic Effects of Ezetimibe on NAFLD

Although the role of dietary fat in the pathogenesis of NAFLD continues to be investigated, evidence from animal studies supports the concept that fat overconsumption plays an important role in the etiology of hepatic steatosis [79]. It has been found that feeding a high-fat diet can induce a significant accumulation of lipids in the liver of animals such as mice and rats [80]. In humans, a large amount of dietary fat could result in the accumulation of triglyceride in the liver, but stable isotope studies found that up to only 15% of lipids accumulated in the liver are derived directly from dietary fat [81, 82]. In contrast, a low-carbohydrate diet, which is otherwise rich in protein and fat, has been used as treatment for NAFLD [83]. Furthermore, long-term overconsumption of fat could increase risk for obesity and insulin resistance, which enhances susceptible to NAFLD [84].

**Figure 7:**
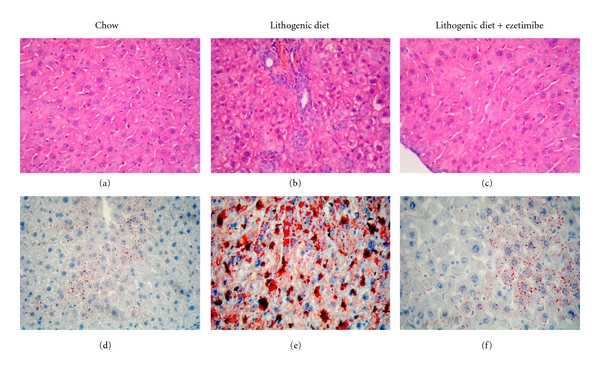
Histological characterization of the hepatic response to ezetimibe in mice fed a chow versus a lithogenic diet for 4 weeks. The liver samples from mice fed with different diets and treated with or without ezetimibe were isolated and subjected to histological analysis. Panels (a)–(c) show representative liver histology with hematoxylin-eosin staining and panels (d)–(f) show Oil Red O staining. (a) and (d) Mice fed with the chow diet. (b) and (e) Mice fed with the lithogenic diet without ezetimibe. (c) and (f) Mice fed eith the lithogenic diet with ezetimibe. The lithogenic diet induced a significant accumulation of triglyceride and cholesteryl ester in the liver as well as hepatocyte damage and inflammation. Interestingly, ezetimibe treatment markedly reduced the accumulation of lipids and prevented hepatic inflammation. Reproduced with modifications and with permission from [52].

Indeed, mice and rats develop hepatic steatosis in response to a high-fat diet and their livers are enlarged and appear grossly pale. Histopathological studies from these livers reveal that hepatocytes are filled with multilocular droplets of varying sizes (Figure 7) [52]. Strikingly, these diet-induced pathological abnormalities are completely absent in livers not only from ezetimibe-treated mice, but also from NPC1L1 deficient mice [79]. In addition, no signs of inflammatory cell infiltration are found in these livers. Hepatic concentrations of both triglyceride and cholesteryl ester are significantly reduced in ezetimibe-treated mice compared with chow-fed control mice [79].

Although a high-fat diet may promote fat accumulation in the liver by simply providing more substrate for triglyceride synthesis, an important mechanism whereby a high-fat diet may drive hepatic steatosis is by causing selective insulin resistance [79, 85]. The increased circulating insulin fails to suppress hepatic gluconeogenesis but can promote hepatic lipogenesis. In contrast, ezetimibe treatment could prevent diet-induced hepatic steatosis, weight gain, and insulin resistance [79]. These alterations are associated with reduced circulating insulin levels, hepatic *de novo* fatty acid synthesis, and hepatic levels of mRNAs for lipogenic genes including glucokinase, an enzyme critical in conversion of glucose to fat. Because elevated blood insulin increases hepatic lipogenic gene expression via transcription factors such as SREBP-1c [16, 86, 87] and glucokinase is an important mediator in this lipogenic pathway [16, 88], ezetimibe treatment may protect against diet-induced hepatic steatosis by reducing hepatic lipogenesis, mostly through preventing diet-induced insulin resistance and the associated hyperinsulinemia. 

Because excessive amounts of cholesterol are lipogenic through activation of LXR by its metabolites [16, 89, 90], reduced intestinal cholesterol absorption by ezetimibe could significantly decrease cholesterol content in the liver. This may prevent diet-induced hepatic steatosis in part by reducing cholesterol-dependent LXR activation in the liver [16].

Nevertheless, ezetimibe treatment indeed plays a significant role in preventing diet-induced fatty liver in animals such as mice and rats; however, its therapeutic effect on NAFLD needs to be further investigated and proven in humans.

## 8. Future Research Directions and Clinical Applications

Ezetimibe is a highly potential and selective cholesterol absorption inhibitor that prevents absorption of cholesterol from dietary and biliary sources by suppressing uptake and transport of cholesterol through the enterocytes. Although there is clear evidence showing that ezetimibe can inhibit cholesterol absorption through the NPC1L1 pathway, careful and systematic studies are needed to confirm whether ezetimibe could reduce intestinal absorption of fatty acids in animal models by direct measurement of their absorption and lymphatic transport and studies need to be undertaken in humans by a balance method of intestinal fatty acid absorption. Because of significantly reduced absorption of intestinal cholesterol and fatty acids, the physical structure of chylomicrons and their metabolism in adipose tissues and liver could be influenced by ezetimibe treatment. To evaluate treatment duration, clinical response rates and the overall cost-benefit analysis on cholesterol gallstones and NAFLD, long-term human studies are needed. Similar to atherosclerosis, the risk for cholesterol gallstone formation and NAFLD increases with dyslipidemia, hyperinsulinemia, obesity, diabetes, sedentary lifestyle, and aging. It is highly likely that the long-term administration of ezetimibe may benefit this group of subjects who could have a high predisposition to cholesterol gallstones and NAFLD.

## Figures and Tables

**Table 1:** Plasma and biliary lipids before (day 0) and at day 30 after ezetimibe treatment in humans (20 mg/day)^a^.

Parameter	Overweight subjects without gallstones		Gallstone patients	
	Before	After	Before	After
BMI (kg/m^2^)	31.5 ± 3.8	31.4 ± 3.4	27.0 ± 2.8	27.1 ± 2.3
Plasma lipid concentrations				
Total Ch (mg/dL)	220 ± 41	168 ± 29^b^	223 ± 32	193 ± 26
LDL Ch (mg/dL)	144 ± 53	99 ± 36	145 ± 26	115 ± 23^b^
HDL Ch (mg/dL)	44 ± 13	37 ± 13	45 ± 11	45 ± 11
TG (mg/dL)	164 ± 88	160 ± 104	166 ± 64	165 ± 76
Biliary lipid compositions of gallbladder biles				
Ch (mole%)	7.4 ± 0.7	6.8 ± 1.9	9.3 ± 1.9	7.2 ± 1.2^b^
PL (mole%)	20.2 ± 2.4	21.8 ± 2.5	19.3 ± 2.8	20.0 ± 3.5
BS (mole%)	72.4 ± 2.9	71.4 ± 3.9	71.4 ± 4.3	72.8 ± 4.2
Ch/PL ratio	0.37 ± 0.03	0.31 ± 0.08	0.48 ± 0.05	0.37 ± 0.06^c^
Ch/BS ratio	0.10 ± 0.01	0.10 ± 0.03	0.13 ± 0.03	0.10 ± 0.02
[TL] (g/dL)	5.3 ± 0.4	5.0 ± 0.9	5.5 ± 0.7	5.3 ± 0.8
CSI	1.2 ± 0.1	1.0 ± 0.2	1.6 ± 0.2	1.3 ± 0.2^b^
CDT (days)	6.4 ± 1.1	10.4 ± 1.1^c^	4.0 ± 1.2	7.0 ± 1.3^c^

^a^Values were determined from overweight subjects without gallstones (*n* = 5) and gallstone patients (*n* = 7).

^b^ *P* < 0.05 and ^c^ *P* < 0.01, compared with before ezetimibe treatment (paired *t* test).

BMI: body mass index; TG: triglycerides; Ch: cholesterol; PL: phospholipids; BS: bile salts; [TL]: total lipid concentrations; CSI: cholesterol saturation index; CDT: crystal detection time.

Reproduced with slightly modifications and with permission from [15].
